# Percutaneous management of acute vascular occlusion after transcatheter aortic valve implantation procedure

**DOI:** 10.1093/ehjcr/ytaf182

**Published:** 2025-04-09

**Authors:** Domingo López Vázquez, Fernando Rueda Núñez, Ramón Calviño Santos, Pablo Piñón Esteban, José Manuel Vázquez Rodríguez

**Affiliations:** Interventional Cardiology, Cardiology, Complexo Hospitalario Universitario A Coruña (CHUAC), As Xubias, 84, PC 15006, A Coruña, Spain; Pediatric Cardiology, Complexo Hospitalario Universitario A Coruña (CHUAC), As Xubias, 84, PC 15006, A Coruña, Spain; Interventional Cardiology, Cardiology, Complexo Hospitalario Universitario A Coruña (CHUAC), As Xubias, 84, PC 15006, A Coruña, Spain; Interventional Cardiology, Cardiology, Complexo Hospitalario Universitario A Coruña (CHUAC), As Xubias, 84, PC 15006, A Coruña, Spain; Interventional Cardiology, Cardiology, Complexo Hospitalario Universitario A Coruña (CHUAC), As Xubias, 84, PC 15006, A Coruña, Spain

## Case description

**Figure 1:**
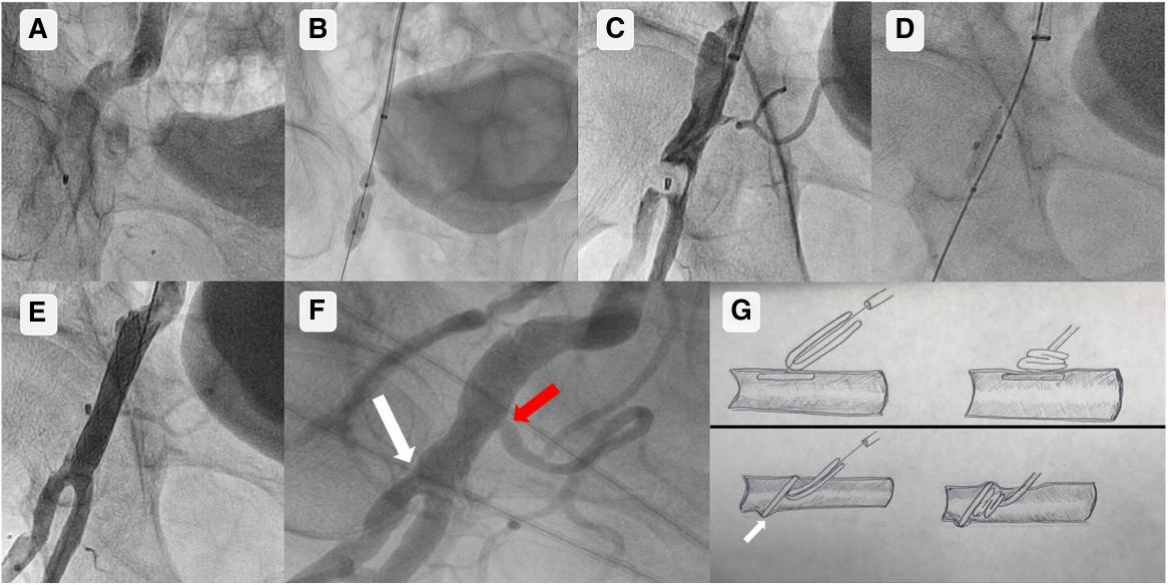
(*A*) Acute femoral artery occlusion due to MANTA failure (white arrow). (*B*) Percutaneous balloon inflation through the anchor of the MANTA device. (*C*) Distal embolization of the MANTA device. (*D*) MANTA being pulled back into the common femoral artery zone. (*E*) Deployment of a self-expandable coated stent, trapping the MANTA against the artery wall. (*F*) Common femoral artery in baseline angiography, with a little gap between the femoral bifurcation (white arrow) and the inferior epigastric artery (red arrow). (*G*) Deployment of MANTA device at the arteriotomy site (top). Intravascular anchor hooked at the origin of the epigastric artery (white arrow) and deploy of the collagen plug through the arteriotomy (bottom).

An 81-year-old male was admitted to our hospital for a transcatheter aortic valve implantation (TAVI) procedure. A balloon expandable aortic prosthesis was implanted through the right femoral artery, without complications. Once the procedure was completed, it was decided to close the right femoral artery with a collagen-based vascular closure device (MANTA) unsuccessful due to acute femoral artery occlusion (*Figure 1A*, Supplementary material online, *Video S1*).

A peripheral angioplasty balloon was inflated in order to restore flow in the limb and a ‘notch’ was observed, probably related to balloon inflation through the anchor of the MANTA device (*Figure 1B*). Unfortunately, these manoeuvres caused a distal embolization of the device (*Figure 1C*, Supplementary material online, *Video S2*). At that moment, a coronary angioplasty balloon was advanced to the superficial femoral artery, and both the balloon and intracoronary guidewire were withdrawn, successfully pulling the embolized device into the common femoral artery zone (*Figure 1D*, Supplementary material online, *Video S3*).

Once the MANTA was placed in the common femoral artery, a peripheral angioplasty balloon was inflated (*Figure 1E*, Supplementary material online, *Video S4*), followed by the deployment of a self-expandable coated stent, trapping the MANTA against the artery wall, waiting for the components of the device to be reabsorbed (see Supplementary material online, *Videos S4* and *S5*).

The MANTA is a device with proven safety and efficacy with a technical success rate of more than 96% described,^1^ without significant differences between this or other vascular closure devices after TAVI.^2^ In this case, our suspicion is that the intravascular deployment of the device is related to the fact that the MANTA anchor may have ‘snagged’ at the origin of the inferior epigastric artery (*Figure 1F*) rather than at the arteriotomy site. Assuming the anchor was against the arteriotomy site, the operator pushed the collagen plug through the arteriotomy into the intravascular space, causing acute arterial occlusion (*Figure 1G*), being this theoretical source of complication postulated with the Angio-Seal device (Terumo Cardiovascular, Tokyo, Japan).^3^

The patient was discharged from the hospital 3 days after the procedure and remains asymptomatic after 48 months follow-up.

## Supplementary Material

ytaf182_Supplementary_Data

## Data Availability

The data underlying this article will be shared on reasonable request to the corresponding author.

## Supplementary material

**Consent:** The patient provided informed consent for data collection and publication. No personal identifying information is provided in this manuscript.

**Funding:** No external funding was received for this clinical case.
